# Long-term abortion and birth rates for 14- to 25-year-olds before and after the implementation of prescription-free emergency contraceptives in Finland

**DOI:** 10.1093/ije/dyag232

**Published:** 2026-10-27

**Authors:** Juha Luukkonen, Olivia McEvoy, Pekka Martikainen, Sini P Laakso, Hanna Remes

**Affiliations:** Helsinki Institute for Demography and Population Health, Faculty of Social Sciences, University of Helsinki, Helsinki, Finland; Max Planck–University of Helsinki Center for Social Inequalities in Population Health, Faculty of Social Sciences, University of Helsinki, Helsinki, Finland; Helsinki Institute for Demography and Population Health, Faculty of Social Sciences, University of Helsinki, Helsinki, Finland; Max Planck–University of Helsinki Center for Social Inequalities in Population Health, Faculty of Social Sciences, University of Helsinki, Helsinki, Finland; Helsinki Institute for Demography and Population Health, Faculty of Social Sciences, University of Helsinki, Helsinki, Finland; Max Planck–University of Helsinki Center for Social Inequalities in Population Health, Faculty of Social Sciences, University of Helsinki, Helsinki, Finland; Helsinki Institute for Demography and Population Health, Faculty of Social Sciences, University of Helsinki, Helsinki, Finland; Max Planck–University of Helsinki Center for Social Inequalities in Population Health, Faculty of Social Sciences, University of Helsinki, Helsinki, Finland; Helsinki Institute for Demography and Population Health, Faculty of Social Sciences, University of Helsinki, Helsinki, Finland; Max Planck–University of Helsinki Center for Social Inequalities in Population Health, Faculty of Social Sciences, University of Helsinki, Helsinki, Finland

**Keywords:** emergency contraceptive, abortions, live births, adolescent, interrupted time series, policy analysis

## Abstract

**Background:**

Public health interventions increasing access to emergency contraceptives (ECs) have been deemed mostly unsuccessful in preventing abortions. However, the assessment of these interventions has mostly ignored potential variation in responses by age at intervention or socio-economic background.

**Methods:**

We used Finnish-population register data to assess annual abortion and live-birth rates for women aged 14–25 years during 1994–2010 by age and parental education. Changes in rates were assessed around the introduction of prescription-free ECs in 2002 with interrupted time-series regressions. We also assessed cohort-wise hazards of abortions and births by age 25 years for cohorts of 1980–94 (aged 8-22 years at the time of intervention).

**Results:**

After the introduction of prescription-free ECs, the increasing annual rates of abortions and live births turned into a clear decline for ages 15–19 years, with a less consistent decline for young people whose parents had low educational attainment. Conversely, the abortion rates continued to increase for ages 20–24 years, with larger increases by lower parental education. Comparing cohorts exposed to the intervention at different ages, we observed lower abortion and live-birth risks by age 19 years among the cohorts who were ≤15 years old at the time of the intervention.

**Conclusion:**

The clear reversal in adolescent abortion rates suggests the prescription-free ECs worked as intended, but the intervention was far less successful among young adults and those with lower socio-economic background. Evaluations of public health interventions need to consider population subgroups.

Key Messages

- We studied whether the long-term abortion and live-birth rates among 14- to 25-year-olds changed in Finland after the implementation of prescription-free emergency contraceptives (ECs) and whether the responses differed by age and socio-economic position.
- We observed that the rising abortion and live-birth rates for ages 15–19 years reversed after the introduction of prescription-free ECs, with some initial fluctuation among those with low parental educational attainment, but, for ages 20–24 years, the abortion rates continued to increase, particularly among those with lower parental educational attainment.
- Birth-cohort comparisons of abortion and live-birth risks suggested that age at the time of intervention mattered, as the declining trends were mostly attributable to the cohorts with earliest exposure to the EC policy change in their lives.

## Introduction

Interventions increasing the availability of emergency contraceptives (ECs) aim to reduce unintended pregnancies and abortions [1, 2], for which adolescents and young adults are especially at risk [3]. While ECs are effective and safe options for preventing unwanted pregnancies [4], timely access to ECs is crucial, as they are effective up until 4 days after unprotected intercourse [5, 6].

Most prior studies do not find any consistent changes in abortion or birth rates after increased access to ECs [1 , 2, 7–13], except for a decrease in adolescent abortions in the USA [14]. Furthermore, a study using longitudinal cross-country data from Europe found no changes in abortion rates after EC-prescription-access lifts, but instead reported increased birth rates, particularly for women aged 25–34 years [13].

Previous studies on how EC-availability interventions affect unintended pregnancies and abortions are mostly limited by small, selected samples or cross-sectional data [2]. Additionally, many studies focus on relatively short-term follow-up periods of 6 months to 2 years, even though such changes might take time to diffuse. Some econometric studies have longer follow-ups and larger population-level data, but still rarely find any notable effects on abortion rates [13–16]. However, even these studies lack a more detailed assessment of trends by age and socio-economic background.

Contraceptive failure and unwanted pregnancies disproportionately affect adolescents and young adults, with potential long-term consequences [3, 17]. Within a life-course framework, adolescence is a sensitive period for the formation of later health behaviours [18]. For example, young age at first abortion is strongly associated with repeat abortions and consequent adverse pregnancy outcomes, such as foetal loss [19]. Studies also show that childbirth at younger ages—in some studies identified as unplanned—is associated with disruptions in the educational and early career trajectories of the mother [20–22] and adverse health outcomes of both the mother and the child [23].

Improved access to EC might be more meaningful for young people, who may lack resources or suffer from greater social stigma attached to unplanned pregnancy, compared with older women. Girls and women with lower socio-economic status experience higher rates of unintended pregnancies and abortions [3, 24–26] and use fewer contraceptives [27] compared with their more socio-economically advantaged counterparts. This suggests that young individuals with lower socio-economic background may be particularly responsive to EC interventions that increase accessibility, but previous research has not considered this possibility.

Using register-based data focusing on women aged 14–25 years and all induced abortions and live births during 1994–2010, our study considers the long-term trends in the age-specific rates of abortions and live births before and after ECs became prescription-free in 2002 in Finland. Induced abortions, which are a proxy for unintended pregnancies, are our primary outcome [28]. Live births are included, to capture alternative pregnancy outcomes potentially affected by EC access. We further consider these abortion and live-birth trends by parental education. Finally, as the intervention increased EC availability at a single point in time, its impact may vary according to the individuals’ age at the time of intervention. We complement the time-series analyses with a cohort approach, comparing the risk of abortion or live birth between cohorts who had access to prescription-free ECs at different ages.

## Methods

### The Finnish context

In Finland, ECs, more specifically levonorgestrel (ATC-code G03AC03), became prescription-free on 1 May 2002 for persons aged ≥15 years. Before this, ECs required a physician’s prescription [12, 29]. A reason behind the prescription-status change was the rising abortion rates for persons <20 years old, which had risen by 43% during 1994–2001 [12]. During the study period, ECs were available from all pharmacies, at ∼15–18 euros per item, and surveys suggest that awareness about ECs was consistently high among adolescents [29].

Apart from the rise in youth abortions, the Finnish abortion rate has mostly been below the EU average throughout the 2000s [30], while the birth rate has been close to the EU average [31]. Induced abortions in Finland required evaluation from at least one physician until 2023. Abortion is a personal choice up until 12 weeks of gestational age and, after that, it requires special permission from a national supervisory authority. Over 90% of induced abortions are performed due to personal circumstances [25]. Currently, almost 99% of all induced abortions are medication abortions, while, in the early 2000s, most induced abortions were procedural [32]. Public healthcare with relatively low patient fees covers primary and specialized healthcare for Finnish residents [33]. Most induced abortions are performed in specialized public healthcare and all healthcare providers are bound by law to register all abortions.

### Data sources and study population

Using pseudonymized personal identifiers, we linked longitudinal population data from Statistics Finland to the national register of induced abortions and the birth register, maintained by the Finnish Institute for Health and Welfare [34–36].

The study population consisted of girls and women aged 14–25 years residing in Finland annually during years 1994–2010. Altogether, the data consisted of 6 463 543 person-years.

### Statistical analyses

We modelled annual trends of abortion and birth rates per 1000 persons before and after the intervention for each age (14–25 years) and for categorical age groups (15–19 and 20–24 years). Time trends were modelled in quadratic form, with the linear time trends provided in Supplementary Figs S1–S3. For abortion rates, we used year 2002 as the structural break, as the new policy was active for most of 2002. For birth rates, we used the year 2003 as a structural break, as most of the children born in 2002 were conceived before the implementation of the prescription-free ECs. The annually aggregated study data for these main analyses are presented in Supplementary Table S1.

Furthermore, we assessed potential socio-economic differences in the trends for age groups 15–19 and 20–24 years. We measured the highest education of either biological parent when the child was aged 14 years (basic or unknown education, secondary education, tertiary education). Education is unknown for a small group of people who received their degrees abroad. The annually aggregated study data, stratified by parental educational attainment, for this analysis are presented in Supplementary Table S2.

Standard age-stratified interrupted time-series models capture age-specific period effects, but cannot assess differences arising from the timing and extent of life-course exposure to the new policy. To address this, we complement the time-series analyses with a comparison of cohorts who had access to prescription-free ECs from different ages. Presumably, those able to purchase prescription-free ECs from an earlier age, before establishing patterns of sexual and health behaviours, exhibit lower risks than those already older at the time of intervention. We estimated hazard ratios of first abortion or first live birth between ages 15–19 and 20–25 years for birth cohorts of 1980–94 using Cox regressions to assess potential deviations from the cohort-by-cohort trends. The reference cohort born in 1987 turned 15 years old in 2002 and was the first cohort to be able to purchase prescription-free ECs from that age. The follow-up started when the individual turned 15 years old (20 years old in the analyses concerning ages 20–25 years). Individuals were censored at death, emigration, at the month when they turned 20 (or 26) years old, or at the respective event of first abortion or live birth.

In a supplementary analysis, we present the annual time trends of abortion and birth rates for all women aged 15–44 years with 5-year age-group intervals to enable comparison with previous studies (Supplementary Fig. S4).

Finally, we did not report formal tests for the slope or level differences because nearly all trends exhibited some change that was statistically significant, but many of these changes were too small to be meaningful.

## Results

Table 1 shows the descriptive statistics for the study population aged 15–25 years during 1994–2010. Both the abortion and birth rates were lower in younger ages, and induced abortions were more common than births for ages 15–18 years. Education-wise, lower parental education was associated with much higher abortion and birth rates at ages 15–19 and 20–24 years.

**Table 1:** Descriptive statistics for the study population.

**Panel A: Women aged 15–25 years during 1994–2010** (Figs 1 and 2)					
Age (years)	*N* (person-years)	*N* abortions	*N* births	Abortion rate	Birth rate
15	543 457	1712	129	3.2	0.2
16	544 510	4095	612	7.5	1.1
17	545 077	6719	2071	12.3	3.8
18	546 272	8417	4941	15.4	9.0
19	544 597	10 086	10 734	18.5	19.7
20	542 687	10 130	18 112	18.7	33.4
21	538 244	9516	23 799	17.7	44.2
22	503 837	8979	29 075	17.8	57.7
23	535 815	8415	34 621	15.7	64.6
24	537 978	8073	41 502	15.0	77.1
25	540 037	7660	49 182	14.2	91.1
Total	5 922 511	83 802	214 778	14.1	36.3

**Panel B: Women aged 15–19 years during 1994–2010 by their parental education at 14 years** (Fig. 3)					
Education	*N* (person-years)	*N* abortions	*N* births	Abortion rate	Birth rate
Basic	317 903	5805	4670	18.3	14.7
Secondary	1 159 377	15 739	9989	13.6	8.6
Tertiary	1 241 251	9353	3433	7.5	2.8
Total	2 718 531	30 897	18 092	11.4	6.7

**Panel C: Women aged 20–24 years during 1994–2010 by their parental education at 14 years** (Fig. 3)					
Education	*N* (person-years)	*N* abortions	*N* births	Abortion rate	Birth rate
Basic	446 552	9942	35 222	22.3	78.9
Secondary	1 131 274	21 109	72 346	18.7	64.0
Tertiary	1 063 368	12 750	33 170	12.0	31.2
Total	2 641 194	43 801	140 738	16.6	53.3

Rates expressed per 1000 person-years.

As shown in Table 1, for the period of 1994–2001, there was a clear increasing trend in the abortion rates among ages 15–19 years, but this development halted after the introduction of prescription-free ECs and turned into a clear decline during the period of 2002–10 (Fig. 1). After the introduction of the prescription-free ECs, it took 8 years for the abortion rates to decrease to the initial levels of 1994. For ages 20–24 years, we observed increases in the abortion rates after the intervention, with highest rates 2 years after the intervention and decline thereafter. Linear trends are presented in Supplementary Fig. S1.

**Figure 1:**
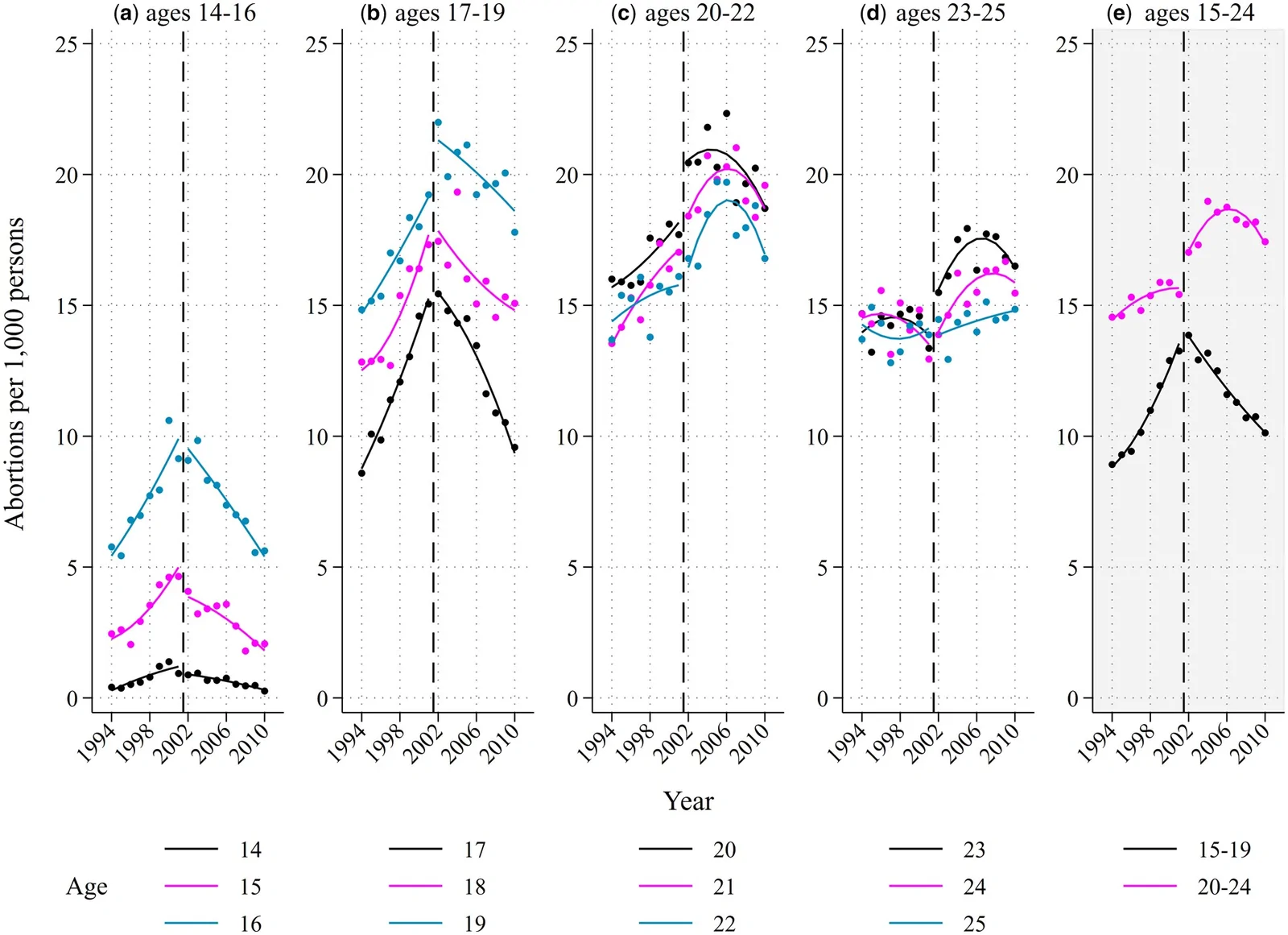
Annual abortion rates per 1000 persons by ages 14–25 years during 1994–2010. Actual rates (small dots) and predicted quadratic time trends, before and onwards from 2002, from an interrupted time-series model (lines). Dashed vertical line represents the time point at which ECs became prescription-free.

Regarding the birth rates, as shown in Fig. 2, for ages 15–20 years, we observed increasing trends 4 years before the intervention and a decrease thereafter (linear trends are presented in Supplementary Fig. S2). The much higher birth rates, especially at ages 22–25 years, drastically decreased in the late 1990s and became more stable around or after the intervention.

**Figure 2:**
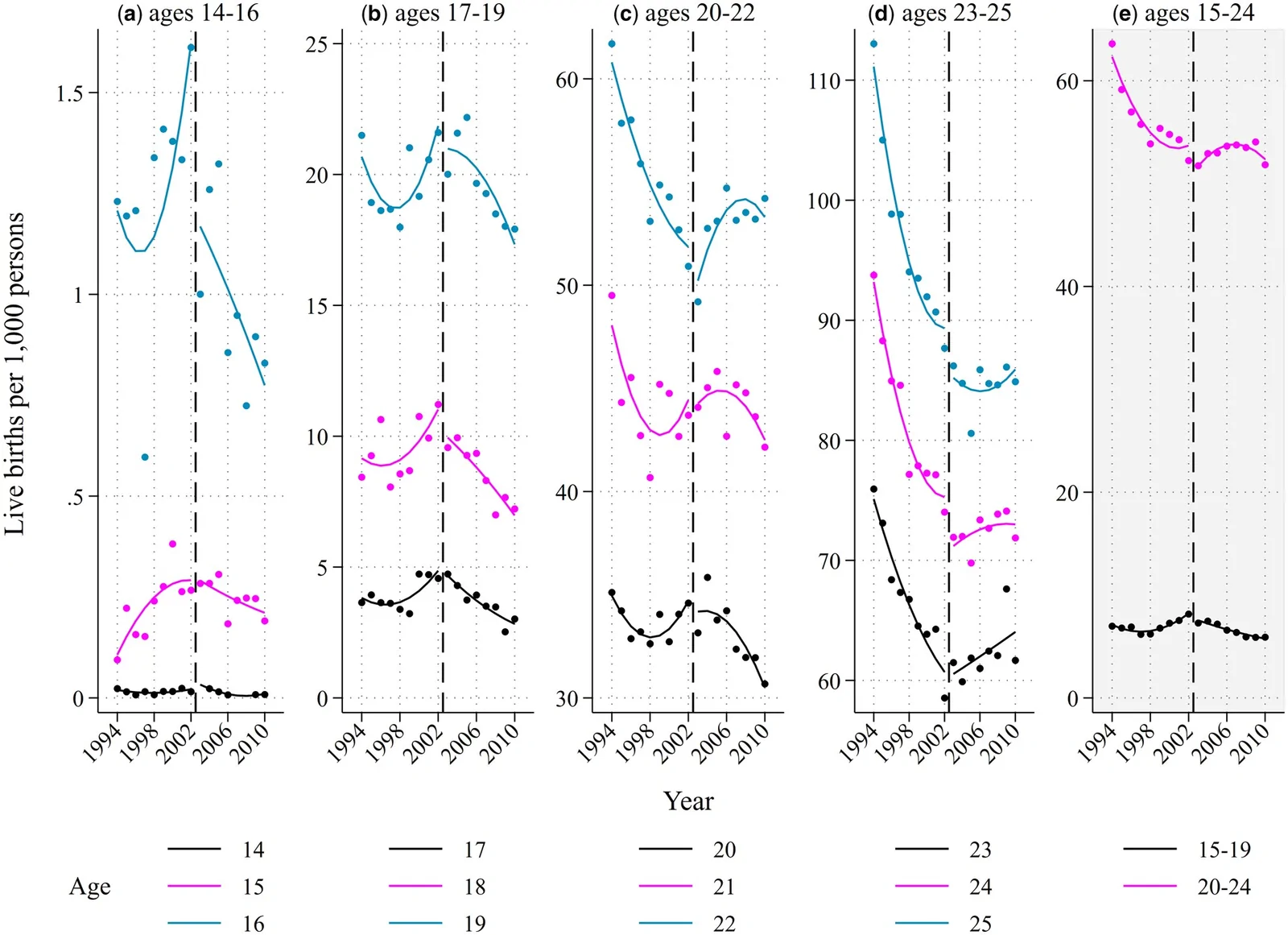
Annual live-birth rates per 1000 persons by ages 14–25 years during 1994–2010. Actual rates (small dots) and predicted quadratic time trends, before and onwards from 2002, from an interrupted time-series model (lines). Dashed vertical line represents the time point at which ECs became prescription-free (for live births, the structural break is 2003, to consider the time from conception to childbirth). Please note the different *y*-axis scales between the panels (a–e).

Figure 3 displays the rates by parental education. The rising trends in abortions and live births at ages 15–19 years halted for all parental education groups and mostly turned into decreasing trends post-intervention. The steepest rise in abortions and births at ages 15–19 years was with persons whose parents had only basic education and this rising trend halted, but fluctuated for several years, turning into a consistent decline. Among those with secondary or tertiary parental education, the curves resembled each other with mostly a level difference. Among the 20- to 24-year-olds, we observed a strong, sustained increase in abortion rates after the implementation of prescription-free ECs among those with lower-educated parents. For the post-intervention birth rates among 20- to 24-year-olds, there was no clear trend change, although a moderate increase in birth rates was observable among women with lower-educated parents. Linear trends are shown in Supplementary Fig. S3.

**Figure 3:**
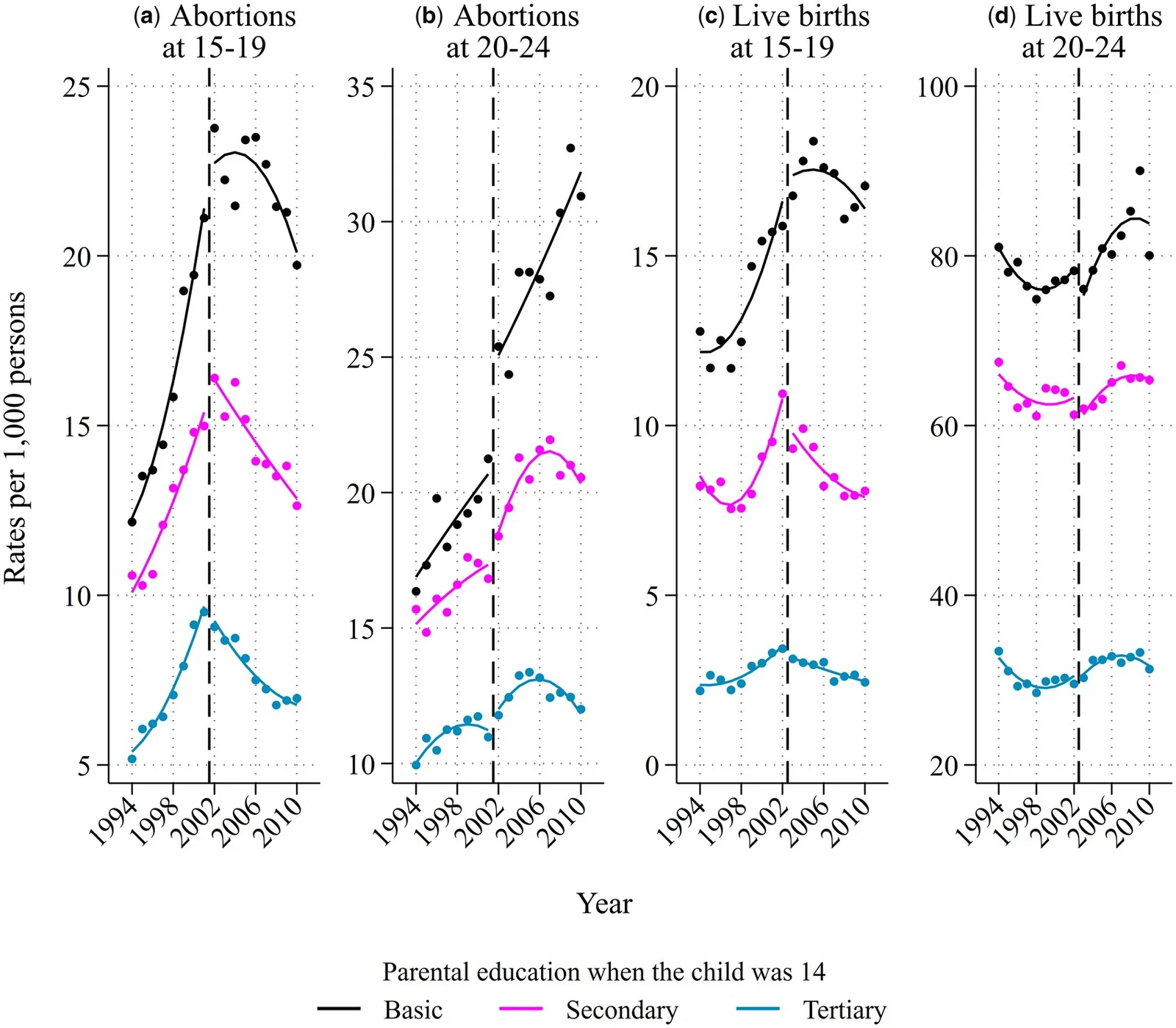
Annual abortion and live-birth rates per 1000 persons by parental education for persons aged 15–19 and 20–24 years old during 1994–2010. Parental education was measured at age 14 years. Actual rates (small dots) and predicted quadratic time trends, before and onwards from 2002, from an interrupted time-series model (lines). Dashed vertical line represents the time point at which ECs became prescription-free. Please note the different *y*-axis scales between the panels (a–d).

Figure 4 shows the survival-analysis results for abortions and live births for birth cohorts of 1980–94. Cohort descriptive statistics are presented in Supplementary Table S3. For the cohorts who were <15 years old (born in 1988–94) when the ECs became prescription-free, the hazard ratios of having an abortion between ages 15 and 19 years were ∼10%–20% lower than those for the reference cohort of 1987, with lowest risks for the more recent cohorts. For the cohorts aged 16–19 years at the time of intervention, the hazard ratios were ∼10% higher, and somewhat lower for those already in their twenties. The hazard ratios for abortions at ages 20–25 years did not notably differ from those of the reference cohort.

**Figure 4:**
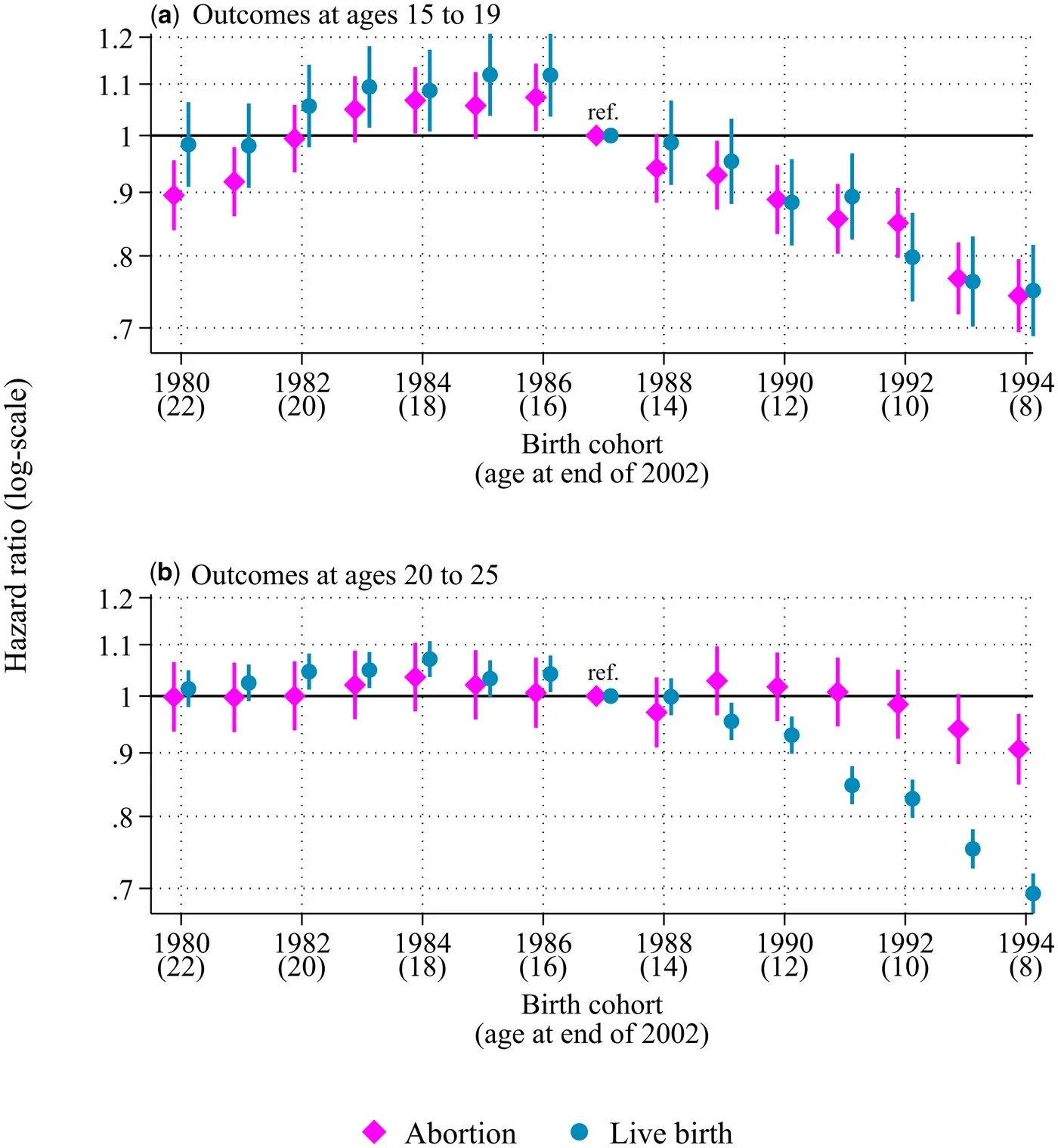
Hazard ratios with 95% confidence intervals for first incidence of induced abortion or first live birth between (a) ages 15–19 years and (b) ages 20–25 years for birth cohorts of 1980–1994 (and age in 2002 when ECs became prescription-free). Reference category: 1987 cohort (turned 15 years old in 2002).

The cohort differences in hazard ratios of live births closely resembled those of abortions, except for ages 20–25 years, where the lower hazard ratios among the cohorts who were <15 years old when the ECs became prescription-free were only observed in births.

Finally, we performed supplementary interrupted time-series analyses for ages 15–44 years with 5-year age categories for comparative purposes (Supplementary Fig. S4). These results suggest that, apart from ages 15–19 years, there were no clear post-intervention changes in the abortion rates. The ongoing decrease in birth rates stalled at ages 20–29 years and turned into an increase at ages 30–34 years, after the prescription-free ECs were introduced.

## Discussion

This study assessed the long-term abortion and live-birth rates before and after the introduction of prescription-free ECs in Finland, with a focus on women aged 14–25 years. For ages 15–19 years, the increasing annual rates of induced abortions and births started declining after the intervention, although there was initial fluctuation among persons with low parental education. Contrasting this, we observed higher abortion rates and slightly higher birth rates among women aged 20–24 years after the intervention, with the largest increases among those with lower parental education. Finally, birth cohorts who had access to the prescription-free ECs when they turned 15 years old had a lower risk of abortion by the age of 19 years and a lower risk of live birth by age 25 years compared with the earlier cohorts who were already ≥15 years at the time of the intervention.

Previous studies assessing abortion rates after interventions that increase access to ECs have mostly found no associations [2, 7–11, 13], with an exception of a decrease in abortions after a policy change that directly affected 18- to 19-years-olds in the USA [14]. A particular strength of our study was the data on single-year age groups and socio-economic position with long observation periods, allowing a detailed assessment of variation across population subgroups. Our findings suggest that both age and socio-economic background were important determinants in how the EC intervention was received. Similarly, the researchers who found reductions in abortion rates among 18- to 19-year-olds in the USA stated that such reductions would have been difficult to observe with aggregate age categories [14]. Also, multi-country comparisons might ignore important country-specific information, such as differences on local healthcare practices potentially influencing the availability of EC products.

Earlier research on EC interventions has explained the lack of impact on abortions with remaining financial barriers or insufficient knowledge about ECs [29]. Our results showing a less consistent decline or even a continued increase in abortion rates among those with lower socio-economic backgrounds support the interpretation that some barriers to access likely remain. Alternatively, increased access to ECs has also been hypothesized to promote risky sexual behaviour or less use of other contraceptives, but the evidence for this is mixed [1, 2, 37], albeit increased unprotected sex [38] and sexually transmitted disease rates have been documented [15, 16]. Furthermore, recent pan-European evidence suggests that birth rates among women aged 24–34 years increased after the over-the-counter availability of ECs was introduced because of less contraceptive-pill use [13]. Our results on increased abortion rates for ages 20–24 years and birth rates for ages 25–34 years (Supplementary Fig. S4) lend further support to this interpretation.

Finally, our cohort comparison showed that individuals who were ≤15 years old (born in 1988–94) when the policy was introduced experienced progressively lower risks of abortion and live birth by age 19 years across successive cohorts, indicating a favourable response among those exposed to prescription-free ECs prior to sexual initiation. Also, partial exposure to the new policy at older ages could explain the stalling of the rising hazard ratios for cohorts of 1983–6.

Sex education in schools might have been a reinforcing factor for increased knowledge about the prescription-free status of ECs among the younger cohorts. Sex education in the school curriculum had increased during the late 1990s and early 2000s [39], although the knowledge of ECs in itself was estimated as rather stable and high among Finnish adolescents around the time of the intervention [29]. Already in 1999, the awareness of ECs was 98% among Finnish 16-year-olds [29]. However, general awareness of ECs does not directly translate into awareness of how to obtain ECs and the survey had a non-negligible nonresponse rate of ∼20% [29].

### Limitations

The register datasets used in our study contain objective measurements for reproductive behaviour encompassing the Finnish population, but, as is often with administrative datasets, we cannot directly observe the exact mechanisms driving these changes. For example, we cannot observe the contraceptive use of individuals, nor trends in sexual activity or behaviour, nor can we assess the role of knowledge towards ECs. However, earlier studies indicate that increased access to ECs increases their use [7, 13, 37, 40].

Furthermore, our findings are descriptive. A control time series not affected by the prescription-status change would strengthen the causal inference [41]. However, apart from gradual increases in sex education from the late 1990s onwards, to the best of our knowledge, there were no other interventions or changes in social conditions coinciding with the implementation of prescription-free ECs. Also, methods of induced abortion have gradually changed during the study period, with medication abortions becoming most common [32], but whether and how the method of abortion would affect abortion rates is unclear.

We used parental education as a measure of socio-economic position because it enabled a comparison across age groups who have not yet attained their own socio-economic position. However, the group whose parents had basic education became smaller and potentially increasingly selective throughout the study period, which may have contributed to the long-term trends, but does not explain the sudden changes in them (Supplementary Table S2).

For birth rates in Finland, there were some changes happening already before the intervention (e.g. at ages 22–25 years), which violates the assumptions of interrupted time-series analysis [42]. The quadratic trends that we employ can partially overcome this, but the potential intervention effect may become indistinguishable from the ongoing trend changes. Overall, changes in birth rates are more difficult to interpret than abortions, given the substantial age-related differences in fertility intentions and pregnancy ambivalence [3]. The sharp decline in births in the late 1990s has previously been interpreted as a change in childbearing, with women not only having children later, but also having fewer children [43].

## Conclusions

After the introduction of prescription-free ECs in Finland, we observed heterogeneous changes in abortion and live-birth rates. While a clear decline in adolescent abortions can be deemed a success, the rates among young adults continued to increase for several years post-intervention, with even less favourable responses among those with lower parental education. Overall, the associations with prescription-free ECs and abortion and birth rates were complex. More detailed long-term analyses are needed to assess how responses to interventions may vary in population subgroups.

## Ethics approval

The study has been approved by the Statistics Finland Board of Statistical Ethics (TK-53-1490-18) and the Social and Health Data Authority Findata (THL/2180/14.02.00/2020). The register data are originally collected for administrative and statistical purposes. The legal basis for processing this kind of personal information is scientific research as stated in the Finnish Personal Data Act (523/1999) and the EU General Data Protection Regulation (GDPR). Use of the data is regulated and permitted by the Act on Secondary use of Social and Healthcare data (552/2019) and the Finnish Statistics Act (280/2004). The funders had no role in the study design, data collection and analysis, decision to publish, or preparation of the manuscript.

## Supplementary Material

dyag232_Supplementary_Data

## Data Availability

The main analyses in the study are performed with annually aggregated data. These datasets are available in Supplementary Tables S1 and S2. However, due to data-protection regulations of the national register-holders, we are unable to make data used in individual-level analyses available to third parties. Interested researchers may contact Statistics Finland. Contact by e-mail: tutkijapalvelut@stat.fi.
