# Supplementary material for: Long-term abortion and birth rates for 14- to 25-year-olds before and after the implementation of prescription-free emergency contraceptives in Finland
Source: Int J Epidemiol. 2026 Oct 27;55(6):dyag232. doi: 10.1093/ije/dyag232 (PMC13712184; doi:10.1093/ije/dyag232)
Supplement: Supplementary file 1 [file dyag232_supplementary_data.docx]

**Supplementary Figure S1** Annual abortion rates per 1,000 persons by ages 14–25 during 1994–2010. Actual rates (small dots) and predicted linear time trends, before and onwards from 2002, from an interrupted time series model (lines). Dashed vertical line represents the time point when emergency contraceptives turned prescription-free.

**Supplementary Figure S2** Annual live birth rates per 1,000 persons by ages 14–25 during 1994–2010. Actual rates (small dots) and predicted linear time trends, before and onwards from 2003, from an interrupted time series model (lines). Dashed vertical line represents the time point when emergency contraceptives turned prescription-free (for live births, the structural break is 2003 to consider the delay in childbirth). Please note the different y-axis scales between the panels A–E.

**Supplementary Figure S3.** Annual abortion and live birth rates per 1,000 persons by parental education for persons aged 15–19 and 20-24 years old during 1994–2010. Parental education was measured at age 14. Actual rates (small dots) and predicted linear time trends, before and onwards from 2002, from an interrupted time series model (lines). Dashed vertical line represents the time point when emergency contraceptives turned prescription-free (for live births, the structural break is 2003 to consider the delay in childbirth). Please note the different y-axis scales between the panels A–D.

**Supplementary Figure S4**. Annual abortion and live birth rates per 1,000 persons by five-year age categories (ages 15-19; 20-24; 25-29; 30-34; 35-40; 40-44) during 1994-2010. Actual rates (small dots), predicted linear time trends (solid lines), and predicted quadratic time trends (dashed lines) from interrupted time series models. Dashed vertical line represents the time point when emergency contraceptives turned prescription-free (for live births, the structural break is 2003 to consider the delay in childbirth). Please note the different y-axis scales between the panels.

**Supplementary Table S1**, part 1/4. Study data. Aggregate numbers of abortions and live births, and shares per 1,000 individuals by year (1994–2010) and age (15–25) in Finland. 14 year-olds are omitted due to very small numbers of abortions and live births.

| year | age | N persons | N abortions | abortions per 1,000 | N live births | live births per 1,000 |
| --- | --- | --- | --- | --- | --- | --- |
| 1994 | 15 | 31,778 | 78 | 2.5 | 3 | 0.1 |
| 1995 | 15 | 31,454 | 82 | 2.6 | 7 | 0.2 |
| 1996 | 15 | 31,800 | 65 | 2.0 | 5 | 0.2 |
| 1997 | 15 | 32,807 | 96 | 2.9 | 5 | 0.2 |
| 1998 | 15 | 33,299 | 118 | 3.5 | 8 | 0.2 |
| 1999 | 15 | 32,594 | 141 | 4.3 | 9 | 0.3 |
| 2000 | 15 | 31,427 | 145 | 4.6 | 12 | 0.4 |
| 2001 | 15 | 30,357 | 141 | 4.6 | 8 | 0.3 |
| 2002 | 15 | 29,952 | 122 | 4.1 | 8 | 0.3 |
| 2003 | 15 | 31,708 | 102 | 3.2 | 9 | 0.3 |
| 2004 | 15 | 31,693 | 108 | 3.4 | 9 | 0.3 |
| 2005 | 15 | 32,663 | 115 | 3.5 | 10 | 0.3 |
| 2006 | 15 | 32,649 | 117 | 3.6 | 6 | 0.2 |
| 2007 | 15 | 33,081 | 91 | 2.8 | 8 | 0.2 |
| 2008 | 15 | 32,305 | 58 | 1.8 | 8 | 0.2 |
| 2009 | 15 | 32,478 | 68 | 2.1 | 8 | 0.2 |
| 2010 | 15 | 31,412 | 65 | 2.1 | 6 | 0.2 |
| 1994 | 16 | 31,698 | 183 | 5.8 | 39 | 1.2 |
| 1995 | 16 | 31,819 | 173 | 5.4 | 38 | 1.2 |
| 1996 | 16 | 31,470 | 214 | 6.8 | 38 | 1.2 |
| 1997 | 16 | 31,834 | 222 | 7.0 | 19 | 0.6 |
| 1998 | 16 | 32,863 | 254 | 7.7 | 44 | 1.3 |
| 1999 | 16 | 33,345 | 265 | 7.9 | 47 | 1.4 |
| 2000 | 16 | 32,629 | 346 | 10.6 | 45 | 1.4 |
| 2001 | 16 | 31,488 | 288 | 9.1 | 42 | 1.3 |
| 2002 | 16 | 30,397 | 276 | 9.1 | 49 | 1.6 |
| 2003 | 16 | 29,985 | 295 | 9.8 | 30 | 1.0 |
| 2004 | 16 | 31,748 | 264 | 8.3 | 40 | 1.3 |
| 2005 | 16 | 31,738 | 258 | 8.1 | 42 | 1.3 |
| 2006 | 16 | 32,716 | 241 | 7.4 | 28 | 0.9 |
| 2007 | 16 | 32,704 | 229 | 7.0 | 31 | 0.9 |
| 2008 | 16 | 33,142 | 224 | 6.8 | 24 | 0.7 |
| 2009 | 16 | 32,393 | 180 | 5.6 | 29 | 0.9 |
| 2010 | 16 | 32,541 | 183 | 5.6 | 27 | 0.8 |
| 1994 | 17 | 32,366 | 278 | 8.6 | 118 | 3.6 |
| 1995 | 17 | 31,732 | 320 | 10.1 | 125 | 3.9 |
| 1996 | 17 | 31,846 | 314 | 9.9 | 116 | 3.6 |
| 1997 | 17 | 31,517 | 359 | 11.4 | 114 | 3.6 |
| 1998 | 17 | 31,874 | 385 | 12.1 | 108 | 3.4 |
| 1999 | 17 | 32,897 | 429 | 13.0 | 106 | 3.2 |
| 2000 | 17 | 33,369 | 487 | 14.6 | 158 | 4.7 |
| 2001 | 17 | 32,674 | 492 | 15.1 | 154 | 4.7 |
| 2002 | 17 | 31,530 | 487 | 15.4 | 144 | 4.6 |
| 2003 | 17 | 30,412 | 450 | 14.8 | 144 | 4.7 |
| 2004 | 17 | 30,022 | 430 | 14.3 | 129 | 4.3 |
| 2005 | 17 | 31,792 | 461 | 14.5 | 119 | 3.7 |
| 2006 | 17 | 31,785 | 428 | 13.5 | 125 | 3.9 |
| 2007 | 17 | 32,775 | 381 | 11.6 | 115 | 3.5 |
| 2008 | 17 | 32,776 | 357 | 10.9 | 114 | 3.5 |
| 2009 | 17 | 33,245 | 350 | 10.5 | 84 | 2.5 |
| 2010 | 17 | 32,465 | 311 | 9.6 | 98 | 3.0 |

**Supplementary Table S1**, part 2/4.

| year | age | N persons | N abortions | abortions per 1,000 | N child births | child births per 1,000 |
| --- | --- | --- | --- | --- | --- | --- |
| 1994 | 18 | 32,713 | 420 | 12.8 | 276 | 8.4 |
| 1995 | 18 | 32,405 | 417 | 12.9 | 300 | 9.3 |
| 1996 | 18 | 31,770 | 411 | 12.9 | 338 | 10.6 |
| 1997 | 18 | 31,880 | 405 | 12.7 | 257 | 8.1 |
| 1998 | 18 | 31,542 | 485 | 15.4 | 270 | 8.6 |
| 1999 | 18 | 31,888 | 523 | 16.4 | 277 | 8.7 |
| 2000 | 18 | 32,913 | 540 | 16.4 | 354 | 10.8 |
| 2001 | 18 | 33,426 | 579 | 17.3 | 332 | 9.9 |
| 2002 | 18 | 32,721 | 571 | 17.5 | 367 | 11.2 |
| 2003 | 18 | 31,561 | 522 | 16.5 | 302 | 9.6 |
| 2004 | 18 | 30,470 | 589 | 19.3 | 303 | 9.9 |
| 2005 | 18 | 30,097 | 482 | 16.0 | 279 | 9.3 |
| 2006 | 18 | 31,876 | 480 | 15.1 | 298 | 9.3 |
| 2007 | 18 | 31,894 | 508 | 15.9 | 265 | 8.3 |
| 2008 | 18 | 32,870 | 478 | 14.5 | 230 | 7.0 |
| 2009 | 18 | 32,889 | 504 | 15.3 | 252 | 7.7 |
| 2010 | 18 | 33,357 | 503 | 15.1 | 241 | 7.2 |
| 1994 | 19 | 31,815 | 472 | 14.8 | 684 | 21.5 |
| 1995 | 19 | 32,697 | 496 | 15.2 | 619 | 18.9 |
| 1996 | 19 | 32,376 | 497 | 15.4 | 603 | 18.6 |
| 1997 | 19 | 31,753 | 540 | 17.0 | 593 | 18.7 |
| 1998 | 19 | 31,852 | 532 | 16.7 | 573 | 18.0 |
| 1999 | 19 | 31,489 | 578 | 18.4 | 662 | 21.0 |
| 2000 | 19 | 31,823 | 573 | 18.0 | 610 | 19.2 |
| 2001 | 19 | 32,867 | 632 | 19.2 | 676 | 20.6 |
| 2002 | 19 | 33,371 | 734 | 22.0 | 721 | 21.6 |
| 2003 | 19 | 32,680 | 651 | 19.9 | 654 | 20.0 |
| 2004 | 19 | 31,553 | 658 | 20.9 | 681 | 21.6 |
| 2005 | 19 | 30,474 | 644 | 21.1 | 676 | 22.2 |
| 2006 | 19 | 30,106 | 579 | 19.2 | 592 | 19.7 |
| 2007 | 19 | 31,907 | 625 | 19.6 | 615 | 19.3 |
| 2008 | 19 | 32,003 | 629 | 19.7 | 592 | 18.5 |
| 2009 | 19 | 32,903 | 660 | 20.1 | 593 | 18.0 |
| 2010 | 19 | 32,928 | 586 | 17.8 | 590 | 17.9 |
| 1994 | 20 | 30,613 | 490 | 16.0 | 1,075 | 35.1 |
| 1995 | 20 | 31,822 | 506 | 15.9 | 1,089 | 34.2 |
| 1996 | 20 | 32,668 | 515 | 15.8 | 1,074 | 32.9 |
| 1997 | 20 | 32,403 | 515 | 15.9 | 1,076 | 33.2 |
| 1998 | 20 | 31,752 | 558 | 17.6 | 1,036 | 32.6 |
| 1999 | 20 | 31,774 | 554 | 17.4 | 1,082 | 34.1 |
| 2000 | 20 | 31,473 | 570 | 18.1 | 1,030 | 32.7 |
| 2001 | 20 | 31,855 | 564 | 17.7 | 1,085 | 34.1 |
| 2002 | 20 | 32,863 | 672 | 20.4 | 1,137 | 34.6 |
| 2003 | 20 | 33,357 | 683 | 20.5 | 1,106 | 33.2 |
| 2004 | 20 | 32,700 | 713 | 21.8 | 1,172 | 35.8 |
| 2005 | 20 | 31,559 | 640 | 20.3 | 1,066 | 33.8 |
| 2006 | 20 | 30,533 | 682 | 22.3 | 1,045 | 34.2 |
| 2007 | 20 | 30,218 | 572 | 18.9 | 978 | 32.4 |
| 2008 | 20 | 32,005 | 629 | 19.7 | 1,023 | 32.0 |
| 2009 | 20 | 32,108 | 650 | 20.2 | 1,026 | 32.0 |
| 2010 | 20 | 32,984 | 617 | 18.7 | 1,012 | 30.7 |

**Supplementary Table S1**, part 3/4.

| year | age | N persons | N abortions | abortions per 1,000 | N child births | child births per 1,000 |
| --- | --- | --- | --- | --- | --- | --- |
| 1994 | 21 | 27,612 | 374 | 13.5 | 1,367 | 49.5 |
| 1995 | 21 | 30,640 | 434 | 14.2 | 1,358 | 44.3 |
| 1996 | 21 | 31,848 | 487 | 15.3 | 1,450 | 45.5 |
| 1997 | 21 | 32,659 | 472 | 14.5 | 1,395 | 42.7 |
| 1998 | 21 | 32,399 | 511 | 15.8 | 1,318 | 40.7 |
| 1999 | 21 | 31,726 | 551 | 17.4 | 1,434 | 45.2 |
| 2000 | 21 | 31,769 | 521 | 16.4 | 1,422 | 44.8 |
| 2001 | 21 | 31,469 | 536 | 17.0 | 1,343 | 42.7 |
| 2002 | 21 | 31,876 | 587 | 18.4 | 1,393 | 43.7 |
| 2003 | 21 | 32,868 | 613 | 18.7 | 1,449 | 44.1 |
| 2004 | 21 | 33,395 | 692 | 20.7 | 1,504 | 45.0 |
| 2005 | 21 | 32,756 | 649 | 19.8 | 1,501 | 45.8 |
| 2006 | 21 | 31,675 | 643 | 20.3 | 1,352 | 42.7 |
| 2007 | 21 | 30,725 | 646 | 21.0 | 1,388 | 45.2 |
| 2008 | 21 | 30,432 | 578 | 19.0 | 1,363 | 44.8 |
| 2009 | 21 | 32,180 | 591 | 18.4 | 1,404 | 43.6 |
| 2010 | 21 | 32,215 | 631 | 19.6 | 1,358 | 42.2 |
| 1994 | 22 | 28,636 | 392 | 13.7 | 1,767 | 61.7 |
| 1995 | 22 | 27,621 | 425 | 15.4 | 1,598 | 57.9 |
| 1996 | 22 | 30,665 | 468 | 15.3 | 1,779 | 58.0 |
| 1997 | 22 | 31,903 | 513 | 16.1 | 1,783 | 55.9 |
| 1998 | 22 | 32,712 | 451 | 13.8 | 1,737 | 53.1 |
| 1999 | 22 | 32,410 | 510 | 15.7 | 1,778 | 54.9 |
| 2000 | 22 | 31,780 | 493 | 15.5 | 1,725 | 54.3 |
| 2001 | 22 | 31,790 | 512 | 16.1 | 1,675 | 52.7 |
| 2002 | 22 | 31,560 | 530 | 16.8 | 1,607 | 50.9 |
| 2003 | 22 | 31,992 | 528 | 16.5 | 1,574 | 49.2 |
| 2004 | 22 | 32,962 | 609 | 18.5 | 1,739 | 52.8 |
| 2005 | 22 | 33,517 | 661 | 19.7 | 1,780 | 53.1 |
| 2006 | 22 | 32,880 | 648 | 19.7 | 1,799 | 54.7 |
| 2007 | 22 | 31,854 | 563 | 17.7 | 1,693 | 53.1 |
| 2008 | 22 | 30,937 | 556 | 18.0 | 1,656 | 53.5 |
| 2009 | 22 | 30,618 | 576 | 18.8 | 1,629 | 53.2 |
| 2010 | 22 | 32,393 | 544 | 16.8 | 1,756 | 54.2 |
| 1994 | 23 | 30,229 | 444 | 14.7 | 2,296 | 76.0 |
| 1995 | 23 | 28,683 | 379 | 13.2 | 2,097 | 73.1 |
| 1996 | 23 | 27,661 | 404 | 14.6 | 1,892 | 68.4 |
| 1997 | 23 | 30,712 | 437 | 14.2 | 2,068 | 67.3 |
| 1998 | 23 | 31,904 | 468 | 14.7 | 2,130 | 66.8 |
| 1999 | 23 | 32,662 | 485 | 14.8 | 2,108 | 64.5 |
| 2000 | 23 | 32,417 | 473 | 14.6 | 2,070 | 63.9 |
| 2001 | 23 | 31,802 | 425 | 13.4 | 2,044 | 64.3 |
| 2002 | 23 | 31,822 | 493 | 15.5 | 1,863 | 58.5 |
| 2003 | 23 | 31,629 | 510 | 16.1 | 1,945 | 61.5 |
| 2004 | 23 | 32,087 | 562 | 17.5 | 1,922 | 59.9 |
| 2005 | 23 | 33,163 | 595 | 17.9 | 2,052 | 61.9 |
| 2006 | 23 | 33,702 | 551 | 16.3 | 2,056 | 61.0 |
| 2007 | 23 | 33,162 | 588 | 17.7 | 2,071 | 62.5 |
| 2008 | 23 | 32,106 | 566 | 17.6 | 1,993 | 62.1 |
| 2009 | 23 | 31,172 | 525 | 16.8 | 2,108 | 67.6 |
| 2010 | 23 | 30,902 | 510 | 16.5 | 1,906 | 61.7 |

**Supplementary Table S1**, part 4/4.

| year | age | N persons | N abortions | abortions per 1,000 | N child births | child births per 1,000 |
| --- | --- | --- | --- | --- | --- | --- |
| 1994 | 24 | 31,270 | 459 | 14.7 | 2,932 | 93.8 |
| 1995 | 24 | 30,284 | 433 | 14.3 | 2,674 | 88.3 |
| 1996 | 24 | 28,710 | 447 | 15.6 | 2,439 | 85.0 |
| 1997 | 24 | 27,722 | 364 | 13.1 | 2,345 | 84.6 |
| 1998 | 24 | 30,736 | 464 | 15.1 | 2,372 | 77.2 |
| 1999 | 24 | 31,879 | 448 | 14.1 | 2,483 | 77.9 |
| 2000 | 24 | 32,703 | 485 | 14.8 | 2,527 | 77.3 |
| 2001 | 24 | 32,436 | 420 | 12.9 | 2,502 | 77.1 |
| 2002 | 24 | 31,839 | 442 | 13.9 | 2,357 | 74.0 |
| 2003 | 24 | 31,934 | 467 | 14.6 | 2,297 | 71.9 |
| 2004 | 24 | 31,721 | 515 | 16.2 | 2,284 | 72.0 |
| 2005 | 24 | 32,225 | 485 | 15.1 | 2,249 | 69.8 |
| 2006 | 24 | 33,347 | 517 | 15.5 | 2,447 | 73.4 |
| 2007 | 24 | 33,944 | 554 | 16.3 | 2,467 | 72.7 |
| 2008 | 24 | 33,442 | 547 | 16.4 | 2,470 | 73.9 |
| 2009 | 24 | 32,371 | 540 | 16.7 | 2,399 | 74.1 |
| 2010 | 24 | 31,415 | 486 | 15.5 | 2,258 | 71.9 |
| 1994 | 25 | 31,961 | 438 | 13.7 | 3,613 | 113.0 |
| 1995 | 25 | 31,280 | 467 | 14.9 | 3,285 | 105.0 |
| 1996 | 25 | 30,307 | 434 | 14.3 | 2,996 | 98.9 |
| 1997 | 25 | 28,725 | 368 | 12.8 | 2,839 | 98.8 |
| 1998 | 25 | 27,744 | 367 | 13.2 | 2,609 | 94.0 |
| 1999 | 25 | 30,723 | 437 | 14.2 | 2,873 | 93.5 |
| 2000 | 25 | 31,881 | 456 | 14.3 | 2,932 | 92.0 |
| 2001 | 25 | 32,713 | 454 | 13.9 | 2,967 | 90.7 |
| 2002 | 25 | 32,493 | 470 | 14.5 | 2,849 | 87.7 |
| 2003 | 25 | 31,921 | 413 | 12.9 | 2,752 | 86.2 |
| 2004 | 25 | 32,044 | 460 | 14.4 | 2,716 | 84.8 |
| 2005 | 25 | 31,854 | 468 | 14.7 | 2,567 | 80.6 |
| 2006 | 25 | 32,380 | 453 | 14.0 | 2,781 | 85.9 |
| 2007 | 25 | 33,563 | 508 | 15.1 | 2,844 | 84.7 |
| 2008 | 25 | 34,209 | 494 | 14.4 | 2,895 | 84.6 |
| 2009 | 25 | 33,655 | 489 | 14.5 | 2,898 | 86.1 |
| 2010 | 25 | 32,584 | 484 | 14.9 | 2,766 | 84.9 |

**Supplementary Table S2**, part 1/2. Study data for persons aged 15-19 and 20-24 years old. Aggregate numbers of abortions and live births, and shares per 1,000 individuals in Finland. Aggregation by year (1994–2010) and maximum parental education at age 14 (basic, secondary, tertiary).

| year | ages | parental education | N persons | N abortions | abortions per 1000 | N child births | child births per 1000 |
| --- | --- | --- | --- | --- | --- | --- | --- |
| 1994 | 15-19 | basic | 30,920 | 376 | 12.2 | 395 | 12.8 |
| 1995 | 15-19 | basic | 28,638 | 387 | 13.5 | 335 | 11.7 |
| 1996 | 15-19 | basic | 26,372 | 361 | 13.7 | 330 | 12.5 |
| 1997 | 15-19 | basic | 24,383 | 352 | 14.4 | 285 | 11.7 |
| 1998 | 15-19 | basic | 22,783 | 361 | 15.8 | 284 | 12.5 |
| 1999 | 15-19 | basic | 21,508 | 408 | 19.0 | 316 | 14.7 |
| 2000 | 15-19 | basic | 20,014 | 389 | 19.4 | 309 | 15.4 |
| 2001 | 15-19 | basic | 18,848 | 398 | 21.1 | 296 | 15.7 |
| 2002 | 15-19 | basic | 17,505 | 416 | 23.8 | 278 | 15.9 |
| 2003 | 15-19 | basic | 16,276 | 362 | 22.2 | 273 | 16.8 |
| 2004 | 15-19 | basic | 14,947 | 321 | 21.5 | 266 | 17.8 |
| 2005 | 15-19 | basic | 14,091 | 330 | 23.4 | 259 | 18.4 |
| 2006 | 15-19 | basic | 13,406 | 315 | 23.5 | 236 | 17.6 |
| 2007 | 15-19 | basic | 12,907 | 293 | 22.7 | 225 | 17.4 |
| 2008 | 15-19 | basic | 12,307 | 264 | 21.5 | 198 | 16.1 |
| 2009 | 15-19 | basic | 11,745 | 250 | 21.3 | 193 | 16.4 |
| 2010 | 15-19 | basic | 11,253 | 222 | 19.7 | 192 | 17.1 |
| 1994 | 15-19 | secondary | 69,806 | 739 | 10.6 | 574 | 8.2 |
| 1995 | 15-19 | secondary | 70,056 | 721 | 10.3 | 568 | 8.1 |
| 1996 | 15-19 | secondary | 69,762 | 741 | 10.6 | 582 | 8.3 |
| 1997 | 15-19 | secondary | 70,074 | 846 | 12.1 | 529 | 7.5 |
| 1998 | 15-19 | secondary | 70,444 | 927 | 13.2 | 533 | 7.6 |
| 1999 | 15-19 | secondary | 70,896 | 971 | 13.7 | 566 | 8.0 |
| 2000 | 15-19 | secondary | 70,652 | 1,046 | 14.8 | 642 | 9.1 |
| 2001 | 15-19 | secondary | 69,722 | 1,045 | 15.0 | 664 | 9.5 |
| 2002 | 15-19 | secondary | 67,925 | 1,114 | 16.4 | 743 | 10.9 |
| 2003 | 15-19 | secondary | 66,945 | 1,022 | 15.3 | 624 | 9.3 |
| 2004 | 15-19 | secondary | 65,985 | 1,074 | 16.3 | 654 | 9.9 |
| 2005 | 15-19 | secondary | 65,840 | 1,000 | 15.2 | 617 | 9.4 |
| 2006 | 15-19 | secondary | 66,440 | 927 | 14.0 | 546 | 8.2 |
| 2007 | 15-19 | secondary | 67,340 | 934 | 13.9 | 571 | 8.5 |
| 2008 | 15-19 | secondary | 66,758 | 902 | 13.5 | 529 | 7.9 |
| 2009 | 15-19 | secondary | 66,089 | 913 | 13.8 | 525 | 7.9 |
| 2010 | 15-19 | secondary | 64,643 | 817 | 12.6 | 522 | 8.1 |
| 1994 | 15-19 | tertiary | 59,464 | 308 | 5.2 | 130 | 2.2 |
| 1995 | 15-19 | tertiary | 61,219 | 371 | 6.1 | 162 | 2.6 |
| 1996 | 15-19 | tertiary | 62,927 | 391 | 6.2 | 158 | 2.5 |
| 1997 | 15-19 | tertiary | 65,094 | 418 | 6.4 | 144 | 2.2 |
| 1998 | 15-19 | tertiary | 67,963 | 480 | 7.1 | 163 | 2.4 |
| 1999 | 15-19 | tertiary | 69,621 | 551 | 7.9 | 203 | 2.9 |
| 2000 | 15-19 | tertiary | 71,288 | 651 | 9.1 | 214 | 3.0 |
| 2001 | 15-19 | tertiary | 72,030 | 685 | 9.5 | 238 | 3.3 |
| 2002 | 15-19 | tertiary | 72,313 | 656 | 9.1 | 248 | 3.4 |
| 2003 | 15-19 | tertiary | 72,894 | 632 | 8.7 | 228 | 3.1 |
| 2004 | 15-19 | tertiary | 74,267 | 649 | 8.7 | 224 | 3.0 |
| 2005 | 15-19 | tertiary | 76,503 | 623 | 8.1 | 226 | 3.0 |
| 2006 | 15-19 | tertiary | 78,897 | 592 | 7.5 | 239 | 3.0 |
| 2007 | 15-19 | tertiary | 81,657 | 591 | 7.2 | 201 | 2.5 |
| 2008 | 15-19 | tertiary | 83,494 | 565 | 6.8 | 218 | 2.6 |
| 2009 | 15-19 | tertiary | 85,483 | 590 | 6.9 | 227 | 2.7 |
| 2010 | 15-19 | tertiary | 86,137 | 600 | 7.0 | 210 | 2.4 |

**Supplementary Table S2**, part 2/2.

| year | ages | parental education | N persons | N abortions | abortions per 1000 | N child births | child births per 1000 |
| --- | --- | --- | --- | --- | --- | --- | --- |
| 1994 | 20-24 | basic | 41,386 | 677 | 16.4 | 3,354 | 81.0 |
| 1995 | 20-24 | basic | 39,201 | 679 | 17.3 | 3,061 | 78.1 |
| 1996 | 20-24 | basic | 37,496 | 742 | 19.8 | 2,972 | 79.3 |
| 1997 | 20-24 | basic | 35,401 | 637 | 18.0 | 2,706 | 76.4 |
| 1998 | 20-24 | basic | 33,475 | 630 | 18.8 | 2,507 | 74.9 |
| 1999 | 20-24 | basic | 31,087 | 598 | 19.2 | 2,363 | 76.0 |
| 2000 | 20-24 | basic | 28,751 | 568 | 19.8 | 2,216 | 77.1 |
| 2001 | 20-24 | basic | 26,496 | 563 | 21.2 | 2,045 | 77.2 |
| 2002 | 20-24 | basic | 24,574 | 624 | 25.4 | 1,923 | 78.3 |
| 2003 | 20-24 | basic | 22,987 | 560 | 24.4 | 1,749 | 76.1 |
| 2004 | 20-24 | basic | 21,787 | 613 | 28.1 | 1,706 | 78.3 |
| 2005 | 20-24 | basic | 20,364 | 573 | 28.1 | 1,647 | 80.9 |
| 2006 | 20-24 | basic | 19,120 | 533 | 27.9 | 1,533 | 80.2 |
| 2007 | 20-24 | basic | 17,793 | 485 | 27.3 | 1,466 | 82.4 |
| 2008 | 20-24 | basic | 16,653 | 505 | 30.3 | 1,420 | 85.3 |
| 2009 | 20-24 | basic | 15,404 | 504 | 32.7 | 1,387 | 90.0 |
| 2010 | 20-24 | basic | 14,577 | 451 | 30.9 | 1,167 | 80.1 |
| 1994 | 20-24 | secondary | 56,506 | 887 | 15.7 | 3,813 | 67.5 |
| 1995 | 20-24 | secondary | 59,228 | 879 | 14.8 | 3,827 | 64.6 |
| 1996 | 20-24 | secondary | 62,504 | 1,005 | 16.1 | 3,882 | 62.1 |
| 1997 | 20-24 | secondary | 65,202 | 1,016 | 15.6 | 4,083 | 62.6 |
| 1998 | 20-24 | secondary | 68,004 | 1,129 | 16.6 | 4,157 | 61.1 |
| 1999 | 20-24 | secondary | 69,042 | 1,216 | 17.6 | 4,447 | 64.4 |
| 2000 | 20-24 | secondary | 69,245 | 1,205 | 17.4 | 4,448 | 64.2 |
| 2001 | 20-24 | secondary | 68,950 | 1,160 | 16.8 | 4,407 | 63.9 |
| 2002 | 20-24 | secondary | 69,218 | 1,273 | 18.4 | 4,242 | 61.3 |
| 2003 | 20-24 | secondary | 69,644 | 1,354 | 19.4 | 4,318 | 62.0 |
| 2004 | 20-24 | secondary | 70,175 | 1,494 | 21.3 | 4,371 | 62.3 |
| 2005 | 20-24 | secondary | 69,992 | 1,434 | 20.5 | 4,417 | 63.1 |
| 2006 | 20-24 | secondary | 69,127 | 1,492 | 21.6 | 4,500 | 65.1 |
| 2007 | 20-24 | secondary | 67,331 | 1,478 | 22.0 | 4,517 | 67.1 |
| 2008 | 20-24 | secondary | 66,382 | 1,370 | 20.6 | 4,351 | 65.5 |
| 2009 | 20-24 | secondary | 65,444 | 1,375 | 21.0 | 4,298 | 65.7 |
| 2010 | 20-24 | secondary | 65,280 | 1,342 | 20.6 | 4,268 | 65.4 |
| 1994 | 20-24 | tertiary | 43,834 | 436 | 9.9 | 1,465 | 33.4 |
| 1995 | 20-24 | tertiary | 46,721 | 511 | 10.9 | 1,452 | 31.1 |
| 1996 | 20-24 | tertiary | 49,855 | 523 | 10.5 | 1,460 | 29.3 |
| 1997 | 20-24 | tertiary | 53,053 | 597 | 11.3 | 1,569 | 29.6 |
| 1998 | 20-24 | tertiary | 56,168 | 629 | 11.2 | 1,601 | 28.5 |
| 1999 | 20-24 | tertiary | 58,469 | 679 | 11.6 | 1,745 | 29.8 |
| 2000 | 20-24 | tertiary | 60,231 | 707 | 11.7 | 1,809 | 30.0 |
| 2001 | 20-24 | tertiary | 61,858 | 679 | 11.0 | 1,872 | 30.3 |
| 2002 | 20-24 | tertiary | 64,070 | 755 | 11.8 | 1,894 | 29.6 |
| 2003 | 20-24 | tertiary | 67,016 | 834 | 12.4 | 2,029 | 30.3 |
| 2004 | 20-24 | tertiary | 68,604 | 909 | 13.2 | 2,220 | 32.4 |
| 2005 | 20-24 | tertiary | 70,296 | 940 | 13.4 | 2,278 | 32.4 |
| 2006 | 20-24 | tertiary | 71,071 | 936 | 13.2 | 2,332 | 32.8 |
| 2007 | 20-24 | tertiary | 71,408 | 888 | 12.4 | 2,289 | 32.1 |
| 2008 | 20-24 | tertiary | 71,947 | 908 | 12.6 | 2,354 | 32.7 |
| 2009 | 20-24 | tertiary | 73,316 | 913 | 12.5 | 2,439 | 33.3 |
| 2010 | 20-24 | tertiary | 75,451 | 906 | 12.0 | 2,362 | 31.3 |

**Supplementary Table S3** Descriptive statistics of the cohort comparison population, birth cohorts 1980-1994

| **Panel A:** Ages 15-19 (Figure 4) | |  |  |  |  |
| --- | --- | --- | --- | --- | --- |
| Birth cohort | N | N first abortion | N first birth | First abortion rate | First birth rate |
| 1980 | 31,428 | 1,808 | 1,284 | 57.5 | 40.9 |
| 1981 | 31,771 | 1,878 | 1,297 | 59.1 | 40.8 |
| 1982 | 32,759 | 2,094 | 1,429 | 63.9 | 43.6 |
| 1983 | 33,270 | 2,254 | 1,502 | 67.7 | 45.1 |
| 1984 | 32,549 | 2,265 | 1,462 | 69.6 | 44.9 |
| 1985 | 31,390 | 2,174 | 1,448 | 69.3 | 46.1 |
| 1986 | 30,287 | 2,139 | 1,397 | 70.6 | 46.1 |
| 1987 | 29,904 | 1,982 | 1,232 | 66.3 | 41.2 |
| 1988 | 31,671 | 1,962 | 1,285 | 61.9 | 40.6 |
| 1989 | 31,644 | 1,939 | 1,236 | 61.3 | 39.1 |
| 1990 | 32,604 | 1,910 | 1,183 | 58.6 | 36.3 |
| 1991 | 32,588 | 1,854 | 1,191 | 56.9 | 36.5 |
| 1992 | 33,013 | 1,860 | 1,073 | 56.3 | 32.5 |
| 1993 | 32,261 | 1,632 | 1,000 | 50.6 | 31.0 |
| 1994 | 32,404 | 1,602 | 981 | 49.4 | 30.3 |
| **Total** | 479,543 | 29,353 | 19,000 | 61.2 | 39.6 |
|  |  |  |  |  |  |
| **Panel B:** Ages 20-25 (Figure 4) | | | | | |
| Birth cohort | N | N first abortion | N first birth | First abortion rate | First birth rate |
| 1980 | 28,563 | 1,891 | 6,328 | 66.2 | 221.5 |
| 1981 | 28,868 | 1,913 | 6,336 | 66.3 | 219.5 |
| 1982 | 29,554 | 1,966 | 6,589 | 66.5 | 222.9 |
| 1983 | 29,878 | 2,024 | 6,686 | 67.7 | 223.8 |
| 1984 | 29,241 | 2,010 | 6,650 | 68.7 | 227.4 |
| 1985 | 28,194 | 1,919 | 6,228 | 68.1 | 220.9 |
| 1986 | 27,211 | 1,825 | 5,968 | 67.1 | 219.3 |
| 1987 | 27,199 | 1,806 | 5,701 | 66.4 | 209.6 |
| 1988 | 28,960 | 1,873 | 6,032 | 64.7 | 208.3 |
| 1989 | 29,123 | 1,981 | 5,785 | 68.0 | 198.6 |
| 1990 | 30,051 | 2,024 | 5,838 | 67.4 | 194.3 |
| 1991 | 30,136 | 2,011 | 5,376 | 66.7 | 178.4 |
| 1992 | 30,701 | 1,987 | 5,288 | 64.7 | 172.2 |
| 1993 | 30,151 | 1,866 | 4,787 | 61.9 | 158.8 |
| 1994 | 30,402 | 1,811 | 4,420 | 59.6 | 145.4 |
| **Total** | 438,232 | 28,907 | 88,012 | 66.0 | 200.8 |
|  |  |  |  |  |  |
| First abortion or birth refers to first induced abortion or live birth occurring at ages 15-19 or 20-25. | | | | | |
| Outcomes happening before age of 15 or 20 are excluded from the counts. | | | | |  |
| Rates expressed per 1,000 persons. | |  |  |  |  |
